# Pancreatic Ductal Adenocarcinoma and Immune Checkpoint Inhibitors: The Gray Curtain of Immunotherapy and Spikes of Lights

**DOI:** 10.3390/curroncol30040293

**Published:** 2023-03-30

**Authors:** Rita Balsano, Valentina Zanuso, Angelo Pirozzi, Lorenza Rimassa, Silvia Bozzarelli

**Affiliations:** 1 Medical Oncology and Hematology Unit, IRCCS Humanitas Research Hospital, Via Manzoni 56, Rozzano, 20089 Milan, Italy; rita.balsano@cancercenter.humanitas.it (R.B.); valentina.zanuso@cancercenter.humanitas.it (V.Z.); angelo.pirozzi@cancercenter.humanitas.it (A.P.); silvia.bozzarelli@cancercenter.humanitas.it (S.B.); 2 Department of Biomedical Sciences, Humanitas University, Via Rita Levi Montalcini 4, Pieve Emanuele, 20072 Milan, Italy

**Keywords:** PDAC, immunotherapy, ICIs, PD-1, PD-L1, CTLA-4, CAR-T cells, vaccines, PARP

## Abstract

Pancreatic ductal adenocarcinoma (PDAC) is a dismal disease with a poor 5-year overall survival rate (~10%). The revolution of immunotherapy in clinical oncology has not substantially changed clinical outcome for patients with PDAC. Despite outstanding efforts, neither immune checkpoint inhibitors (ICIs) alone, nor in combination with chemotherapy or targeted therapies have shown encouraging results. This failure mirrors the lack of knowledge about the real key players of immune system senescence and the complexity of the tumor microenvironment in PDAC. However, some hope can be derived from PARP-inhibitor combinations, vaccines, and CAR-T-cells therapy. In this review, we comprehensively summarize the latest updates about the use of ICIs in PDAC, focusing on clinical evidence and ongoing studies highlighting explanations for the failure of immunotherapy and possible solutions.

## 1. Introduction

Pancreatic ductal adenocarcinoma (PDAC) is a sneaky and aggressive tumor with an increasing incidence in both sexes worldwide. Even though the current knowledge of PDAC has improved, its treatment still represents one of the most significant challenges in solid tumors. Moreover, the prognosis for patients with advanced disease remains dismal, with a 5-year overall survival (OS) rate of less than 10% [1]. In these patients, the current standard-of-care treatment is represented by palliative systemic chemotherapy with FOLFIRINOX (folinic acid, 5-fluorouracil, irinotecan, and oxaliplatin) or gemcitabine and nab-paclitaxel [2,3].

In the last decade, immunotherapy has revolutionized the treatment algorithm for cancer. It has been incorporated into the therapeutic strategy of various solid malignancies, often constituting the best therapeutic choice since first-line treatment [4]. This change is primarily attributed to the advent of immune checkpoint inhibitors (ICIs) [5].

Immune evasion is an established hallmark of cancer. The revolutionary findings by the group of Allison et al. at the MD Anderson Cancer Center showed this feature might be mainly ascribed to the activation of membrane proteins expressed on both immune cells (natural killer cells, T lymphocytes, B lymphocytes) and malignant cells [6,7]. The most studied are programmed cell death protein 1 (PD-1), its ligand (PD-L1), and cytotoxic T-lymphocyte antigen 4 (CTLA-4). The PD-L1/PD-1 axis is crucial for immune tolerance, preventing excessive stimulation of the immune system, and, thus, avoiding the onset of autoimmune disorders.

**Figure 1:**
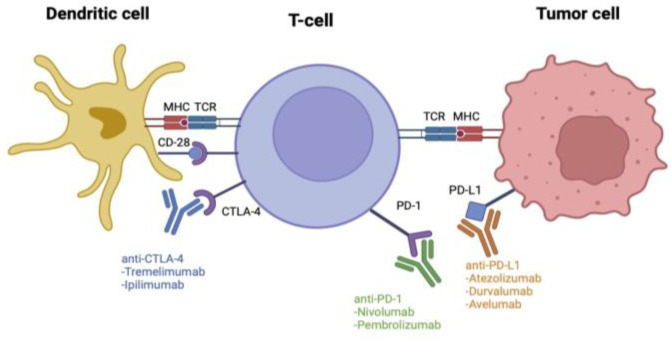
The mechanism of action of ICIs. Abbreviations: ICIs, immune checkpoint inhibitors; MHC, major histocompatibility complex; TCR, T-cell receptor; CD-28, cluster of differentiation 28; CTLA-4, cytotoxic T-lymphocyte antigen 4; PD-1, programmed cell death protein 1; PD-L1, programmed cell death protein ligand 1.

In the context of PDAC, binding PD-1 to its ligand inhibits molecular signals, typically promoting the activation of effector lymphocytes [8]. CTLA-4 receptor is a decoy competitor of the cluster of differentiation 28 receptor (CD28), highly expressed on T-Cell effectors for B7-1 and B7-2 ligands of cancer cells [9]. B7-1 or B7-2 binding to CD28 normally results in a costimulatory activating signal required for CD8-T cell effectors functioning [10]. CTLA-4 binding escapes the above costimulatory, disabling an effective cell-mediated immune response. Therefore, a sensitive balance exists between levels of CD28 and CTLA-4 expression in T cells, bringing the final decision to either attack or save cancer cells [11]. Although effector T cells can recognize cancer antigens binding the major histocompatibility complex class I (MHC-I), a costimulatory signal is needed to induce cell death effectively. The lack of costimulatory signals due to the PD-1/PD-L1 and CTLA-4 hyperactivation makes the capacity of T cells to target cancer useless. The final result is the development of immune tolerance against cancer. Based on these mechanisms, monoclonal antibodies selectively targeting PD-1/PD-L1 and CTLA-4 were developed to unlock the immune system and enhance the immune response [12]. Figure 1 describes the mechanism of action of ICIs.

A progressive understanding of the above mechanism showed that the higher the expression of PD-1 and/or PD-L1, the better the antitumor response. However, studies showed that while this is true for some cancer types but not for other solid tumors like pancreatic cancer. Moreover, the rate of PD-L1 expression is highly variable (19–62.5%) in PDAC, and its expression is significantly associated with poor prognosis [13]. This suggests the interplay of other influencing factors like cytokines, subsets of different immune cells (medullary dendritic cells, regulatory T cells, hostile physical extracellular environment), and different immune checkpoints, which might play a role in immune escape [14].

In PDAC, tumor growth is advantaged by a strongly immunosuppressive context, which has promoted numerous studies investigating its role and potential therapeutic implication. In particular, targeting the recruitment of immunosuppressive mediators can be used as a strategy to modify the hypoxic and desmoplastic tumor microenvironment. In fact, PDAC is considered an immunologically “cold” tumor with low mutational burden, which represents a strong rationale for the application of immunomodulatory approaches in order to enhance immune response against cancer cells [11].

**Figure 2:**
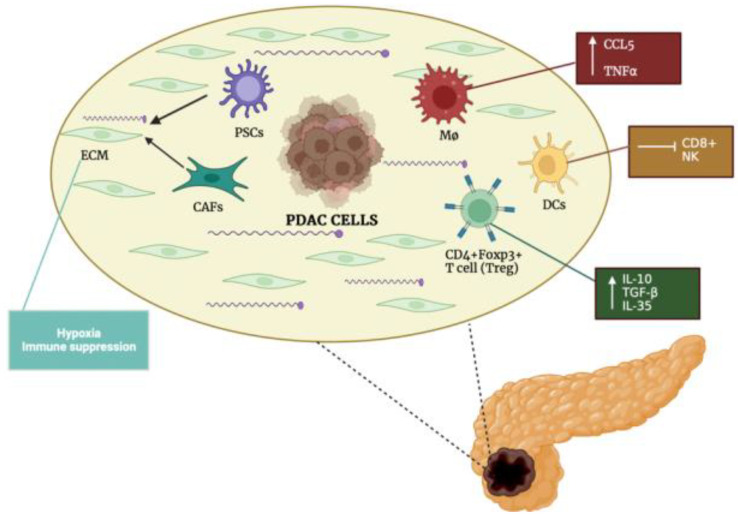
The immune landscape in PDAC. Abbreviations: PDAC, pancreatic ductal adenocarcinoma; CAFs, cancer-associated fibroblasts; PSCs, pancreatic stellate cells; ECM, extra-cellular matrix; CCL5, CC motif chemokine ligand 5; TNFα, tumor necrosis factor alfa; Mø, macrophages; CD4+Foxp3+T cell Treg, regulatory T cell; IL-10, interleukin 10; IL-35, interleukin 35; TGF-β, transforming growth factor beta; DCs, dendritic cells; NK, natural killer.

Figure 2 represents the complex interplay between PDAC cells, immune cells, and other cells within the tumor microenvironment. Cancer-associated fibroblasts (CAFs) and pancreatic stellate cells (PSCs) are key factors in the production of collagen and other components of the extra-cellular matrix (ECM), causing desmoplasia and immune suppression. In addition, stimulated macrophages (Mø) release CC motif chemokine ligand 5 (CCL5) and tumor necrosis factor alfa (TNFα) which are immunosuppressive cytokines involved in the immune modulation of T cells. Moreover, CD4+Foxp3+T cells (T reg) induce interleukin 10 (IL-10), interleukin 35 (IL-35), and transforming growth factor beta (TGF-β), which contribute to deepen suppression of immune response. Dendritic cells (DCs) prevent cytotoxic CD8+ and natural killer (NK) cells from invading the tumor and triggering an anti-cancer response.

One of the most innovative immunotherapeutic strategies is represented by adoptive CAR (chimeric antigen receptor) T-cell therapy, which consists of patients’ genetically modified T-cells expansion to recognize a specific tumor antigen [15]. Moreover, cancer vaccines represent a promising new therapeutic approach [16]. Unfortunately, immunotherapeutic and immunomodulatory treatments have shown only limited efficacy in PDAC [17]. In this review, we will shed light on what has already been explored and the ongoing studies, providing our personal view of the most interesting future perspectives.

## 2. Methods

The two main topics, “ICIs” and “PDAC”, were codified by combining free text and MeSH database vocabulary keywords to search on the Pubmed.gov database by the advanced search tool. A restriction was made for papers, including the keywords in the corresponding title and abstract. The “ICIs” term was encoded with the following string:

“Immune Checkpoint Inhibitors” [Mesh] OR “PD-L1 Inhibitor*” [tiab] OR “Immune Checkpoint Blocker*” [tiab] OR “Immune Checkpoint Blockade” [tiab] OR “Programmed Death Ligand 1 Inhibitor*” [tiab] OR “Cytotoxic T-Lymphocyte-Associated Protein 4 Inhibitor*” [tiab] OR “PD-1 Inhibitor*” [tiab].

The “PDAC” term was encoded as follows:

“Carcinoma, Pancreatic Ductal” [Mesh] OR “Pancreatic Ductal Carcinoma” [tiab] OR “Pancreatic Ductal adenocarcinoma” [tiab] OR “PDAC” [tiab] OR “Pancreatic Cancer” [tiab] OR “Pancreas Duct-cell Carcinoma” [tiab] OR “Pancreatic Neoplasm*” [tiab]. The above strings were bundled with the “AND” Boolean logical operator. The search was filtered out to the last five years and to the English language, and produced 195 results overall.

Abstracts presented at main international oncology conferences in the last five years were retrieved by manually checking.

### 2.1. Immune Checkpoint Inhibitors

#### 2.1.1. ICI Monotherapy

Ipilimumab, an anti-CTLA-4 monoclonal antibody (mAb), was the first ICI tested in PDAC. In a phase II trial, ipilimumab monotherapy at a dose of 3 mg/kg every 4 weeks administered to 27 patients showed no objective response. Of note, 20 patients had metastatic disease and received previous gemcitabine-based regimens, whereas seven patients had locally advanced disease. Furthermore, ipilimumab was associated with grade 3 or higher immune-related adverse events (irAEs), experienced by 11.1% of patients, mainly represented by colitis, encephalitis, and hypophysitis [18]. Disappointing results also came from a phase II open label study evaluating tremelimumab, another CTLA-4 inhibitor, in different cohorts of patients with selected advanced solid tumors. In detail, in a cohort of 20-gemcitabine- or 5-fluorouracil-pretreated metastatic PDAC patients, 90% (n = 18) had disease progression with a median OS of 4.0 months (95% CI, 2.8–5.1). As with ipilimumab, 55% of patients experienced grade 3 or higher treatment-related adverse events (trAEs) and the most common was mainly represented by diarrhea [19]. Similarly, anti-PD-L1 and anti-PD-1 antibodies failed to demonstrate efficacy in PDAC patients. Anti-PD-L1 antibodies BMS-936559 and MPDL3280A and the anti-PD-1 pembrolizumab failed to show any durable response in patients diagnosed with advanced previously treated PDAC [20,21]. In a phase II trial testing the anti-PD-L1 durvalumab, no objective responses were seen in any of the 32 patients enrolled. TrAEs occurred in 6% of patients, mainly represented by fatigue, diarrhea, and pruritus [22]. Even though these trials suggest the lack of efficacy of ICIs in PDAC, a remarkable exception is represented by tumors with a deficient mismatch repair system (MMR) that is directly involved in error repair during DNA replication. When the system is defective, mutation collection in small repetitive elements defines a condition of microsatellite instability (MSI). As a result, the increased number of neoantigens enhance inflammatory cytokines expression and T cells activation, favoring cancer cells sensibility to immunotherapy [23,24].

Interestingly, MSI-high patients are more likely to respond significantly to anti-PD-1 drugs due to the increased rate of missense mutations arising from the loss of function of mismatched repair proteins known as MutS protein homologue 2 (MSH2), MutS homologue 6 (MSH6), MutL homolog 1, and PMS1 homologue 2 (PMS2). However, the prevalence of MSI-high (MSI-H) status among patients with PDAC is extremely low, ranging from 0.8% to 2% [25]. Nevertheless, the prognosis and survival are better for MSI-H compared to MSI-low (MSI-L) patients treated with ICIs, suggesting the potential role of MSI-H status as a predictor of response to immunotherapy also in PDAC [26]. Pembrolizumab received the United States Food and Drug Administration (US FDA) approval for patients with MSI-H tumors regardless of histology [27]. In a phase II trial, pembrolizumab monotherapy showed durable clinical efficacy in a cohort of non-colorectal cancer patients, including four patients with MSI-H PDAC who received at least two lines of prior standard chemotherapy. In this cohort the median PFS was 5.4 months (95% CI, 3-not estimable [NE]), whereas the median OS was not reached (NR) [28]. In the phase II KEYNOTE-158 trial, 22 previously treated patients with MSI-H PDAC received single-agent pembrolizumab, with an overall response rate (ORR) of 18.2% and a median OS and progression-free survival (PFS) of 4.0 months (95% CI, 2.1–9.8) and 2.1 months (95% CI, 1.9–3.4), respectively. 14.6% of patients had grade 3 or higher trAEs, with one patient experiencing grade 5 pneumonia. Moreover, 9.4% of patients discontinued treatment because of trAEs [29].

#### 2.1.2. ICI Combinations with Other ICI or Targeted Therapy

Since ICI monotherapy demonstrated limited efficacy for patients with PDAC, subsequent trials focused on their combination. Tremelimumab was evaluated in association with durvalumab in a phase II, two-stage trial as a second-line treatment. The combination was administered in 32 patients, showing an ORR of 3.1%, higher than 0% reached with durvalumab monotherapy, which was administered in 33 patients. 22% of patients receiving the combination treatment experienced trAEs of grade 3 or higher, including diarrhea, autoimmune hepatitis, muscular weakness, and myositis. However, the combination did not reach the prespecified efficacy threshold of an ORR greater than 25%, preventing further studies of this combination in PDAC [22]. More recently, other combinations of ICIs and targeted therapies failed to show any relevant potential in PDAC patients. In details, emactuzumab, a colony-stimulating factor-1 receptor (CSF1R) associated with atezolizumab, was tested in a phase Ib study in 221 previously treated patients with advanced solid tumors. The ORRs were 9.8% and 12.5% in treatment-naïve and previously ICI-treated advanced solid tumor patients, respectively. The most common grade 3 or higher trEAs were fatigue, rash, and increased aspartate aminotransferase (AST). Unfortunately, we cannot derive definitive data about the efficacy of this combination because the subgroup analysis of PDAC patients was missing [30]. Finally, the combination of avelumab, another anti-PD-L1 mAb, with binimetinib, a mitogen-activated protein kinase (MEK) inhibitor, was tested in a phase Ib/II study in patients with previously treated metastatic PDAC, failing to show encouraging results; consequently, its development was stopped [31].

#### 2.1.3. ICIs and Chemotherapy

In order to increase the quality and number of neoantigens to try to turn the “cold” TME into a “hot” one, investigators embraced combining immunotherapy with cytotoxic drugs. Several preclinical models documented the synergy of this combination, showing increased priming and activation of T cells, thus promoting immune response [32]. In a dose-escalation phase Ib study, the combination of ipilimumab and gemcitabine was tested in 21 patients with previously treated PDAC and showed a median PFS and OS of 2.8 (95% CI, 1.6–4.8) and 6.9 (95% CI, 2.6–9.6) months, respectively. The ORR was 14%, with two patients experiencing partial response (PR) and five patients maintaining stable disease (SD). Grade 3 or higher trAEs were observed in 19% of the patients (diarrhea and increased AST/alanine aminotransferase (ALT)). Interestingly, no grade 3 or higher endocrine dysfunction, cutaneous toxicities, colitis, or pneumonitis were observed; this is probably attributed to the association of ipilimumab to chemotherapy. However, the addition of ipilimumab to gemcitabine did not demonstrate more effectiveness than published data about gemcitabine alone, showing similar ORR [33]. Likewise, the combination of tremelimumab with gemcitabine in chemotherapy-naïve patients showed a median OS of 7.4 months (95% CI, 5.8–9.4), with two patients achieving PR and seven patients maintaining SD. This treatment combination was well tolerated, with a relatively low grade 3 or higher toxicity rate, represented mainly by asthenia and nausea. Only one patient developed a severe trAE (diarrhea) [34]. Pembrolizumab plus gemcitabine and nab-paclitaxel were evaluated in a first-line phase Ib/II study showing a PFS of 9.1 months (95% CI, 4.9–15.3) and an OS of 15.0 months (95% CI, 6.8–22.6), with a low rate of grade 3 AEs (53%), mainly represented by hematological toxicities [35].

On the contrary, first-line nivolumab plus gemcitabine and nab-paclitaxel showed inconsistent results, and the safety profile did not allow further investigation [36,37]. Moreover, nivolumab plus FOLFIRINOX showed an ORR of 32.3% and a median OS of 13.4 months (90% CI, 10.9–15.2) in treatment-naïve patients with metastatic PDAC [38]. Lastly, the combination of gemcitabine, nab-paclitaxel with or without durvalumab, and tremelimumab was evaluated in the first-line setting in a recent phase II study. However, there was no significant difference in median OS between chemoimmunotherapy and chemotherapy alone (9.8 versus 8.8 months; HR 0.94; 90% CI, 0.71–1.25; *p* = 0.72). Similarly, median PFS was not significantly different between the two arms (5.5 versus 5.4 months; HR 0.98; 90% CI, 0.75–1.29; *p* = 0.91). The rate of AEs was similar in both groups, with grade 3 or higher experiencing fatigue, a thromboembolic event, and sepsis representing the most frequent ones [39].

#### 2.1.4. ICIs and PARP-Inhibitors

Some preclinical studies suggested that the combination of poly ADP ribose polymerase (PARP) inhibitors and ICIs could be effective in patients with PDAC [40]. Maintenance treatment with niraparib plus nivolumab or niraparib plus pembrolizumab was evaluated in a phase Ib/II trial in 91 patients with platinum sensitive PDAC, irrespective of the BRCA status. The primary endpoint of 6-month PFS was met in the niraparib plus ipilimumab group (59.6%). In contrast, the combination of nivolumab plus niraparib showed an inferior result (6-month PFS of 20.6%), favoring the hypothesis of better efficacy of anti-CTLA-4 over anti-PD-1 in this context. In the ipilimumab plus niraparib group, median PFS and median OS were 8.1 months (95% CI, 5.5–10.6) and 17.3 months (95% CI, 12.5–22.2), respectively. Regarding tolerability, 50% of patients receiving ipilimumab plus niraparib had grade 3 or higher AEs, mainly fatigue, anemia and hypertension. On the other hand, nivolumab plus niraparib was better tolerated with a lower rate of trAEs (22%) [41]. Three phase II trials were designed and are ongoing to test the combination of pembrolizumab and olaparib in previously treated patients. Of note, the NCT05093231 trial is assessing this combination in patients with high tumor mutational burden (TMB-High; >4 mutations/Mb) tumors, while the others (NCT04548752, NCTNCT04666740) are assessing the combination efficacy regardless of TMB value. In addition, the same combination is under investigation as a maintenance treatment after multi-agent low-dose first-line chemotherapy with gemcitabine, nab-paclitaxel, capecitabine, cisplatin, and irinotecan (GAX-CI) (NCT04753879).

#### 2.1.5. ICIs and Other Combinations of Particular Interest

The highly immunosuppressive TME is considered a barrier to ICI efficacy. Therefore, the tumor stroma and its elements have been evaluated as a potential treatment target, presuming a direct involvement in tumor progression. The transforming growth factor β (TGF-β), a multifunctional cytokine produced by a variety of cells within the TME, is directly involved in fibrosis and immune evasion, as well as the regulation of cells within TME. This cytokine was evaluated as a potential treatment target in patients with advanced PDAC. In detail, its combination with ICIs showed preliminary efficacy in different solid tumors [42]. In a phase II study, the combination of anti-type 1 TGF-β receptor galunisertib and durvalumab was tested in 42 patients previously treated for metastatic or locally advanced disease and reached a DCR of 25% with one PR and seven SD, with a low rate (16.7%) of high-grade AEs. However, efficacy seemed limited by the low number of tumor antigens [43]. In this setting, the daNIS-1 study, an ongoing phase II trial, is testing the addition of NIS793, a TGF-β inhibitor, to gemcitabine and nab-paclitaxel with or without the anti-PD-1 spartalizumab in metastatic chemo-naïve patients [44].

Similarly, the combination of pembrolizumab and pegylated recombinant human hyaluronidase (PEGPH20) is being tested in an ongoing phase II trial in heavily pre-treated patients. However, its use needs attention because it can increase the pressure within the TME, including blood vessels, with a high risk of thromboembolic events [45]. Other combinations were evaluated in patients with metastatic disease with disappointing results. Particularly, durvalumab plus epacadostat (indoleamine 2,3-dioxygenase 1 [IDO1] inhibitor) was studied in patients previously treated for locally advanced or metastatic disease with no objective response, even though the combination was well tolerated [46]. Pembrolizumab plus the focal adhesion kinase (FAK) inhibitor defactinib and gemcitabine was investigated in patients with metastatic disease that was refractory or stable after at least 4 months of gemcitabine and nab-paclitaxel, demonstrating a DCR of 80% and 70%, respectively [47]. An exception is represented by the combination of the C-X-C motif chemokine receptor 4 (CXCR4) inhibitor BL-8040, pembrolizumab, and liposomal irinotecan and 5-fluorouracil administered with leucovorin. The confirmed promising ORR was 32% and the median DCR was 77% [48]. Lastly, the addition of radiation therapy (RT) to ICIs is supposed to enhance the creation of new tumor antigens, thus favoring PDAC sensibility to immunotherapy [49]. In a phase II study, patients with previously treated metastatic PDAC received ipilimumab, nivolumab, and 8 Gy of RT at cycle two. The ORR was 13.6% with one CR and 2 PRs. However, seven out of 22 patients did not receive any dose of RT because of early disease progression [50]. Since there is no clear evidence of the therapeutic advantage of RT and ICIs, several trials exploring this combination are ongoing, and results are awaited.

Among other ongoing trials of particular interest, a recruiting phase I trial (NCT03970252) is testing the combination of nivolumab with standard chemotherapy (FOLFIRINOX) as a neoadjuvant regimen for 3–6 cycles in patients with resectable and borderline resectable PDAC. Of note, the study comprises a sub-analysis comparing subsets of immune cell infiltrates and IFN-γ signaling activation at baseline and at the time of surgery or tumor progression to identify immune signatures related to treatment response.

Moreover, an emerging field of interest is the combination of ICIs and drugs involved in autophagy. In detail, pancreatic cancer cells use the natural process of autophagy to escape immune surveillance down-regulating MHC-I [51]. Chloroquine, a known inhibitor of autophagy mechanisms, was tested in PDAC in combination with first-line chemotherapy with gemcitabine and nab-paclitaxel, showing no efficacy [52]. Nevertheless, multiple combination strategies including ICIs and autophagy inhibitors, especially in KRAS-mutant PDAC, are under investigation [53]. Ongoing trials are listed in Table 1.

### 2.2. CAR-T-Cell Therapy

In the era of precision medicine, the cancer-immune landscape has attracted considerable attention. To date, several ongoing therapeutic strategies are aimed at eliciting cellular immunity against tumors. CAR-T-cell therapy is an innovative treatment based on collecting T cells genetically modified to express an antigen-binding domain able to recognize and attack cancer cells [54]. Promising preclinical studies also demonstrated CAR-T-cell therapy’s potential role in PDAC but also highlighted peculiar challenges in this neoplasm. Of note, the most recent and surprising success story is the regression of visceral metastases (ORR 72%) in a patient receiving autologous T-cell receptor (TCR)–engineered T cells targeting mutant KRAS G12D pancreatic cells. In addition, the reported response seems to be long-lasting (6 months since the first radiological evidence), which is impressive compared with standard chemotherapy regimens approved in the same setting [55]. Despite the successful clinical experience with TCR–engineered T cells, clinically meaningful case reports for CAR-T cells achieving similar results are still lacking, highlighting the need for a comprehensive analysis of the underlying limits of immune cell therapy. Several challenges need to be addressed to fully implement this strategy, including the presence of dense stroma, disabling factors like immunosuppressive cytokines (interleukin 6 [IL-6], TGF-β), or the prevalence of immunosuppressive immune cell types (T-helper 17 [Th17] cells, myeloid-derived suppressor cells [MDSCs], suppressive T reg interfering with CAR-T cells, and TCR–engineered T cells action) [15]. In addition, the presence of the same target in both cancer cells and healthy cells could cause unexpectedly high toxicity. Several antigens have been studied as targets among the five generations of CARs developed until now.

CD133 is a transmembrane glycoprotein highly expressed in many cancers, such as hepatocellular carcinoma and PDAC. In a phase I study (NCT02541370), CD133-directed CAR-T cells were studied in patients with solid tumors, including seven patients affected by PDAC. Results showed two PRs, three SDs, two PDs out of seven patients, and a 40% tumor reduction for 4 months in a patient with multiple metastases at diagnosis [56].

Mesothelin is a protein and potential target detected in PDAC. A phase I trial (NCT01897415) explored mesothelin-targeted CAR-T cells in six patients with PDAC who did not respond to previous chemotherapy. Although it was well-tolerated with no severe AEs, the best response was SD reported in two patients with a duration of 5.4 and 3.8 months [57]. Similar results were demonstrated in a phase I clinical trial (NCT001935843) studying human epidermal growth factor receptor 2 (HER2), a transmembrane glycoprotein overexpressed in PDAC. Among 11 participants, there were two patients who achieved SD for 5.3 and 8.3 months. The treatment was not well tolerated due to acute infusion-related febrile syndrome [58].

Claudin 18.2 (CLDN18.2) is a tight-junction protein overexpressed in gastric and pancreatic tumors. In a phase I trial, CAR-CLDN18.2 T-cell infusion was administered to 12 patients (of which five with chemo-naïve PDAC), showing a good safety profile and an ORR of 33.3% [59].

The epidermal growth factor receptor (EGFR) is a transmembrane protein known to activate malignant pathways in several tumors. EGFR-directed CAR-T cells were investigated in a phase I trial, enrolling 16 patients with stage IV PDAC. Treatment showed only moderate efficacy as 50% of patients exhibited SD, and two reported PD. In addition, two patients experienced grade 3 treatment-related lung toxicity [60].

Since clinical trials investigating CAR-T-cell therapy have shown only moderate efficacy, future studies are needed to improve this treatment strategy and overcome potential hurdles limiting its efficacy in the PDAC subset [61]. Firstly, identifying the ideal antigen is difficult considering the complexity and heterogeneity of PDAC that, like many solid tumors, does not express specific antigens, resulting in a limited homing of CAR-T cells [61,62]. In addition, the TME of PDAC consisting of different cell types, such as fibroblasts, immune cells, and proteins, plays a role in suppressing the immune response and limiting CAR-T-cell therapy potential benefits. In this direction, different studies are exploring the approach of locoregional delivery of CAR-T-cell therapy into the tumor (NCT01373047, NCT02416466) [54] and also multi-targeting CAR-T cells [63].

### 2.3. Vaccines

Another treatment strategy is represented by vaccines, emerging as innovative immunotherapies, also evaluated in combination with chemotherapy. One of the best-investigated vaccine strategies is granulocyte-macrophage colony-stimulating factor (GM-CSF)-allogeneic pancreatic tumor cells (GVAX). Since 2001, GVAX has been explored in several settings of PDAC, including post-surgical and metastatic settings, alone or in combination with cyclophosphamide. Unfortunately, only modest efficacy was shown in all these trials, especially in patients who reported an induction of mesothelin-specific CD8+ T cells [64]. Recently, the results of the phase III PILLAR study investigating the role of the hyperacute pancreas algenpantucel-L (HAPa) vaccine in locally advanced PDAC have been published. In the trial, the standard arm of cytoreductive therapy with FOLFIRINOX or gemcitabine/nab-paclitaxel was compared to the experimental arm, where the HAPa vaccine was added to standard chemotherapy. Unfortunately, the study failed to demonstrate superiority in OS and PFS that were similar in both arms (14.9 and 14.3 months for OS [HR 1.02; 95% CI, 0.66–1.58; *p* = 0.98]; 13.4 and 12.4 months for PFS [HR 1.33; 95% CI, 0.72–1.78; *p* = 0.59]) [65].

GV1001 is a 16-amino acid peptide vaccine derived from the reverse-transcriptase-subunit of telomerase (hTERT), an overexpressed enzyme in PDAC [66]. Due to its intrinsic activity of inducing CD4 clones and CD8 T cells [67], it has been studied in association with chemotherapy. A phase III trial evaluated 1062 patients with locally advanced or metastatic PDAC randomized in three arms: chemotherapy alone, chemotherapy with sequential GV1001, or chemotherapy combined with the vaccine. Unfortunately, the trial was negative, and median OS was not statistically significantly different between the chemotherapy group and the sequential chemoimmunotherapy group (7.9 versus 6.9 months; HR 1.19; 98.25% CI, 0.97–1.48; *p* = 0.05) or the concurrent chemoimmunotherapy group (7.9 versus 8.4 months; HR 1.05; 98.25% CI, 0.85–1.29; *p* = 0.64) [66].

Another vaccine strategy is represented by the human leukocyte antigen class A HLA-A*2402-restricted KIF20A-derived peptide investigated in the VENUS-PC phase II trial. Patients with locally advanced or metastatic PDAC received the vaccine in combination with epitope-peptides targeting vascular endothelial growth factor receptors 1 and 2 (VEGFR1-2) and gemcitabine, without HLA-A stratification. A subsequent analysis was performed between HLA-A*2402-matched and -unmatched group. The trial did not show any statistically significant difference in OS between the two groups (*p* = 0.456) [68].

CRS-207 is a live, attenuated listeria monocytogenes strain that expresses the tumor-associated mesothelin and can induce innate and mesothelin-specific cell-mediated immunity. It was studied in a phase II trial (ECLIPSE), exploring its efficacy in combination with cyclophosphamide and GVAX (arm A) versus CRS-207 alone (arm B) versus physician’s choice of single-agent chemotherapy (arm C) in pre-treated patients. However, the trial failed to demonstrate a significant difference from standard chemotherapy. In heavily pre-treated patients, median OS was 3.7 (arm A) versus 5.4 (arm B) versus 4.6 months (arm C) showing no significant difference between the three arms (HR 1.17; 95% CI, 0.84–1.64) [69].

## 3. Discussion

As outlined in previous chapters, the considerable efforts to test the efficacy of ICIs in PDAC have yielded disappointing results. Several possible causes for this failure have been identified.

Primarily, pancreatic neoplasms are weakly immunogenic and often present a low TMB and a low antigen expression, considered potential predictors of the efficacy of ICIs. This could explain the relative efficacy of ICIs only in MSI-H PDACs that are, on the contrary, naturally enriched in antigen presentation.

Indeed, no subgroups of patients or predictive biomarkers for immunotherapy have been identified, excluding MSI-H status.

Secondly, as already reported, the TME of pancreatic cancer consists primarily of desmoplastic cells, and only a small part (5–10%) includes pancreatic cancer cells. This dense tangle of complementary cells creates a physical barrier and increases blood vessel pressure, hindering drug delivery.

In addition, there are many other factors, such as the microbiome, inflammatory cells, and immune infiltrate, that are the topic of several preclinical studies and whose role in influencing possible sensitivity to immunotherapy in PDAC is still under investigation. Even though it was supposed that PDAC tissue was not properly in close contact with microbial populations, recent evidence suggests that it harbors species that play a crucial role on TME and on response to ICIs. In fact, it has been demonstrated that some species as bifidobacterium adolescentis, enterococcus faecium and collinsella aerofaciens suppress human T reg, undermining therapeutic ICIs efficacy [70]. Moreover, some preclinical studies in mice found that depletion of oral microbial flora enhances anticancer response in PDAC [71].

A recent review analyzed several studies including patients treated with ICIs in different settings where immunotherapy is the standard of care and identified several confounding factors such as age, alcohol consumption, and lifestyle. Although exhaustive studies in PDAC are lacking, it is possible to assume that the same confounding factors may play a role in decreasing efficacy of ICIs in PDAC patients who often have comorbidities like diabetes, alcohol consumption, and obesity as risk factors [72].

In addition, the weight of histology in predicting immunotherapy response is still unclear. Current evidence suggests different PD-L1 expressions in rare pancreatic cancer histologies, with a higher expression detected in squamous cell carcinoma, potentially predicting a better response to immunotherapy than PDAC. Of note, 83% of squamous cell pancreatic cancer (PASC) expressed PD-1 vs. 11% of PDAC in a study collecting biopsy samples from 50 patients with pancreatic cancer. However, the overall tumor proportion score (TPS) showed a low expression level [73] and the number of PASC patients included in clinical trials is still low. Therefore, category 1 evidence is not available yet.

Nevertheless, there are glimpses of possible trends on which it might be interesting to concentrate future efforts. One of these comes from the encouraging findings from Reiss et al. with the combination of niraparib plus ipilimumab in patients with platinum-sensitive advanced PDAC. The results of this study create opportunities to explore new combinations of ICIs and PARP inhibitors and the potential role for non-cytotoxic maintenance therapies. However, it is yet to be determined why niraparib plus nivolumab failed to achieve the same advantage in PFS reached by niraparib plus ipilimumab, underlying a pivotal role played by CTLA-4 in inhibiting cell-mediated immunity. A reasonable explanation might be the higher CTLA-4 mRNA expression in T-regulatory cells of pancreatic cancer patients and higher CTLA-4 expression at the time of disease progression [74].

Nonetheless, ongoing trials persist in testing PD-1 inhibitors more than CTLA-4-directed strategies.

In addition, this study reflects a possible role of platinum sensitivity as a predictive factor of response to PARP inhibitors associated with ICIs regardless of BRCA status and could represent a turning point in treatment development strategies for patients with PDAC.

Moreover, combining ICIs with standard chemotherapy could improve clinical outcome and represents a still active research topic aiming at overcoming the microenvironment barrier responsible for the immunosuppressive context and chemoresistance. A recently exploratory study presented at American Society of Clinical Oncology (ASCO) Gastrointestinal Cancers Symposium (ASCO GI) 2023 investigated the combination of camrelizumab, an anti-PD-1 agent, with nab-paclitaxel and apatinib, a VEGFR2 inhibitor, as first-line treatment in metastatic PDAC [75]. The results of this single-arm study showed antitumor activity with an ORR of 16.7% and a DCR of 91.7%, supporting the combination of ICI with standard chemotherapy as a potential future strategy. Another phase I study presented at ASCO GI 2023 tested gemcitabine, nab-paclitaxel, pembrolizumab, and SEA-CD40, an IgG1 mAb against CD40 in the metastatic PDAC setting. This combination showed a manageable safety profile and favorable activity with a median PFS of 7.4 months (95% CI, 5.6–9.0) [76].

Finally, new insights can come from novel pathway tested in combination with ICIs, among which the most interesting could be the autophagy pathway.

## 4. Future Directions and Take-Home Messages

There is an urgent need to find new therapeutical strategies for PDAC, which remains one of the most aggressive cancers with a high mortality rate. The hallmark of PDAC is characterized by a dense, desmoplastic, and immunosuppressive stroma that confers intrinsic resistance to both chemotherapy and immunotherapy. Even though immunotherapy has shown encouraging results in many solid cancers, its role remains controversial in PDAC. In fact, ICIs alone or in combination, as well as the most recent CAR-T cells and vaccines did not meet the expected results in terms of efficacy and survival improvement. Therefore, further studies should investigate the factors that represent an obstacle and underlies the resistance to these therapeutic strategies. Moreover, the combination of immunomodulatory approaches with standard chemotherapy should be studied, carefully evaluating the potential side effects of their application. Lastly, the identification of specific predictive biomarkers could help clinicians to identify patients whose tumors are more likely to respond to immunotherapy. Research in PDAC treatment is rapidly increasing and will hopefully lead to more personalized therapies for patients in the future.

## 5. Conclusions

In conclusion, moderate efficacy has been shown from immunotherapeutic approaches, but there is still no effective immunotherapy strategy for patients with PDAC. A deeper understanding of the mechanisms underlying immunomodulation could allow, in future studies, to overcome chemoresistance and natural barriers which are responsible for the limited efficacy of ICIs in PDAC. Further efforts are needed to better investigate the promising role of vaccines and CAR-T-cell therapy in PDAC.

## Figures and Tables

**Table 1:** Ongoing trials of Immunotherapy in patients with PDAC.

Title	Identifier	Phase	Stage	Status
Modulation of the Gut Microbiome with Pembrolizumab Following Chemotherapy in Resectable Pancreatic Cancer	NCT05462496	Pilot study	Resectable PDAC	Not yet recruiting
Study of Pembrolizumab with or Without Defactinib Following Chemotherapy as a Neoadjuvant and Adjuvant Treatment for Resectable Pancreatic Ductal Adenocarcinoma	NCT03727880	Phase II	Resectable PDAC	Recruiting
Pilot Study With CY, Pembrolizumab, GVAX, and IMC-CS4 (LY3022855) in Patients with Borderline Resectable Adenocarcinoma of the Pancreas	NCT03153410	Pilot	Borderline resectable PDAC	Active, not recruiting
Lenvatinib and Pembrolizumab Maintenance Therapy for the Treatment of Patients of Advanced Unresectable Pancreatic Cancer	NCT04887805	Phase II	Unresectable PDAC	Recruiting
Serial Measurements of Molecular and Architectural Responses to Therapy (SMMART) PRIME Trial	NCT03878524	Phase Ib	Unresectable, locally advanced PDAC or mPDAC	Recruiting
Study With CY, Pembrolizumab, GVAX, and SBRT in Patients with Locally Advanced Pancreatic Cancer	NCT02648282	Phase II	Locally advanced PDAC	Active, not recruiting
Pembrolizumab and XL888 in Patients with Advanced Gastrointestinal Cancer	NCT03095781	Phase Ib	Locally advanced and mPDAC	Active, not recruiting
A Pilot Study to Assess Changes in Tumor Biology Following Second-line Treatment with Pembrolizumab Plus Lenvatinib in Patients With Advanced Pancreatic Ductal Adenocarcinoma	NCT05273554	Pilot	mPDAC	Recruiting
Safety Study of SEA-CD40 in Cancer Patients	NCT02376699	Phase I	mPDAC	Active, not recruiting
Personalized Peptide Vaccine in Treating Patients with Advanced Pancreatic Cancer or Colorectal Cancer	NCT02600949	Phase I	mPDAC	Recruiting
Pembrolizumab In Combination with Debio 1143 In Pancreatic and Colorectal Advanced/Metastatic Adenocarcinoma (CATRIPCA)	NCT03871959	Phase I	mPDAC	Active, not recruiting
Epacadostat, Pembrolizumab, and CRS-207, With or Without CY/GVAX Pancreas in Patients with Metastatic Pancreas Cancer	NCT03006302	Phase II	mPDAC	Active, not recruiting
Testing the Addition of Pembrolizumab, an Immunotherapy Cancer Drug to Olaparib Alone as Therapy for Patients with Pancreatic Cancer That Has Spread With Inherited BRCA Mutations	NCT04548752	Phase II	mPDAC	Recruiting
Pembrolizumab and CXCR4 Antagonist BL-8040 in Treating Patients With Metastatic Pancreatic Cancer	NCT02907099	Phase IIb	mPDAC	Active, not recruiting
Neoadjuvant Folfirinox Combined with Pembrolizumab Followed by Surgery for Patients With Resectable Pancreatic Cancer	NCT05132504	Phase II	mPDAC	Recruiting
Pembrolizumab With Olaparib as Combined Therapy in Metastatic Pancreatic Cancer	NCT05093231	Phase II	mPDAC	Not yet recruiting
Study of CRS-207, Pembrolizumab, Ipilimumab, and Tadalafil in Metastatic Pancreatic Cancer	NCT05014776	Phase II	mPDAC	Recruiting
BXCL701 and Pembrolizumab in Patients with Metastatic Pancreatic Ductal Adenocarcinoma(EXPEL PANC)	NCT05558982	Phase II	mPDAC	Not yet recruiting
A Study of Pembrolizumab and Olaparib for People with Metastatic Pancreatic Ductal Adenocarcinoma and Homologous Recombination Deficiency or Exceptional Treatment Response to Platinum-Based Therapy	NCT04666740	Phase II	mPDAC	Recruiting
Multi-agent Low Dose Chemotherapy GAX-CI Followed by Olaparib and Pembro in Metastatic Pancreatic Ductal Cancer.	NCT04753879	Phase II	mPDAC	Recruiting

Abbreviations: PDAC: pancreatic ductal adenocarcinoma; GVAX: granulocyte-macrophage colony-stimulating factor (GM-CSF)-allogeneic pancreatic tumor cells; CY: cyclophosphamide; CXCR4: C-X-C motif chemokine receptor 4; GAX-CI: gemcitabine, nab-paclitaxel, capecitabine, cisplatin, and irinotecan.
